# Gender disparities in Australia’s specialist digital health workforce: a cross-sectional study on education and occupation

**DOI:** 10.1186/s12913-025-13274-8

**Published:** 2025-08-28

**Authors:** Salma Arabi, Kerryn Butler-Henderson, Kathleen Gray, Clair Sullivan, Karen Day

**Affiliations:** 1 School of Nursing, Paramedicine, and Healthcare Sciences, Charles Sturt University, Wagga Wagga, NSW Australia; 2 Centre for Digital Transformation, University of Melbourne, Melbourne, Australia; 3 Queensland Digital Health Centre, The University of Queensland, Brisbane, Australia; 4 School of Population Health, University of Auckland, Auckland, New Zealand

**Keywords:** Gender, Workforce, Digital health, Digital health workforce, Education, Professional development, Health services

## Abstract

**Background:**

Digital transformation is rapidly changing healthcare delivery, which is changing the functions and responsibilities of the health workforce. The specialist digital health workforce support the frontline clinical workforce through the management of health data and information systems. Gender disparity in this new configuration of the healthcare workforce can undermine organisational aims, workforce wellbeing, equitable care, population health, patient experience, and economic sustainability.

**Research aim:**

The aim of this paper was to identify the nature of existing gender disparities and potential strategies for fostering gender equity in Australia’s specialist digital health workforce.

**Methods:**

The 2023 Australian Specialist Digital Health Workforce Census incorporated gender survey questions from the Telstra Health Understanding Gender Diversity in Australia’s Digital Health Sector Special Report 2021-22 for the first time. These data were statistically analysed, examining if there was a difference between women and men in: (1) Education, (2) Professional development, (3) Occupation, and (4) Workforce intention.

**Results:**

There were 857 valid responses, of which 595 (69.43%) respondents identified as woman. Women were less likely than men to have a formal digital health qualification. They were less likely to occupy higher paid roles, where both had formal qualifications. Women have fewer opportunities for mentoring and leadership training than men. Men indicated more senior roles than women, and more men were healthcare practitioners than women. More men than women aimed for senior management roles. Both valued work-life balance in employment. Barriers to career progression included age, financial constraints, outside of work responsibilities.

**Discussion:**

Gender disparities are a workforce issue in the Australian digital health landscape. Women are underrepresented in digital health roles, particularly in technology-related roles and analytics. Women have fewer opportunities for leadership training, which is particularly notable as they occupy fewer leadership roles and fewer aspire to leadership roles. Equitable care should be considered one aspect of equity within the workforce. Policy interventions, mentorship and networking, educational reform, and changes in organisation culture are suggested strategies for balancing gender in the digital health specialist workforce.

## Background

Digital transformation is rapidly reshaping the health workforce, as described in analyses around the world [1–3]. The transformation requires the support of a specialist workforce to support the frontline clinician in safe and responsible use of technology and data. The primary function of the specialist digital health workforce is to manage the technology, systems, platforms, and infrastructure services needed to extract value from data, information and knowledge within the health sector [4]. Their roles and responsibilities may include analyzing, designing, developing, implementing, maintaining, managing, operating, evaluating, and/or governing aspects of digital health [4]. As expectations build for stronger accountability and transparency in the digital transformation of health, recognition for this new professional specialization, and self-identification with it, are emerging.

The new specialist digital health professional workforce is evolving through the convergence of roles in the health workforce that previously were distinct, including clinical professionals, information technology (IT) professionals, health administrators, biomedical engineers, project managers, and others. So far, this new workforce is an amalgam of quite varied educational qualifications and industry certifications, occupational classifications and professional cultures. From a workforce planning perspective, it is important to ensure access to coherent career pathways and inclusive work environments, in order to grow and sustain a skilled workforce in this specialization.

Gender disparity in the health workforce has the potential to undermine all of its aims [5], not only worker well-being, but also equitable care, population health, patient experience and economic sustainability. In the Australian science, technology, engineering, and mathematics (STEM) workforce, from which some digital health specialists are drawn, gender disparity is known to be an issue: 37% of students in STEM-related university courses are women, but only 15% of STEM-qualified jobs are held by women, and proportionately fewer women in STEM work at senior management levels [6]. In the health workforce, from which other digital health specialists are drawn, women predominate, holding 74% of roles, mostly in nursing and midwifery [7]. Historically, these are highly feminized workforces, yet they are positioned low in status and value in the health hierarchy, connected to notions of ‘care’ and ‘women’s work’. Overall rates of participation do not signify parity in the health workforce; other key factors are stereotyping, employment conditions, remuneration, and career development opportunities [8].

Little is known about whether the emerging specialist digital health workforce offers recognized and rewarding roles regardless of gender, or conversely whether it poses gender-based barriers to entry and progression. A clearer picture of the current situation can help health workforce planners and employers to avert workforce shortages, and can promote gender diversity among prospective workers, in a workforce that is increasingly important to delivering value from the digital transformation of health.

## Methods

The objective of this paper is to identify existing gender disparities to inform potential strategies for fostering gender equity in Australia’s specialist digital health workforce, based on data from a national workforce census. The research question guiding this study is: *What gender disparities exist in Australia’s specialist digital health workforce?* The Specialist Digital Health Workforce Census (the Census) was developed from 2016–2018 through a rigorous Delphi process [9]. Many of the demographic items use the same meta-data items as the Australian Bureau of Statistics Household Census [10], with the other questions informed by other workforce studies and the group of experts in the Delphi study. In April 2021, a study was undertaken prior to the 2021 census to validate the questions. In 2023, the census incorporated the gender survey questions from the Telstra Health Understanding Gender Diversity in Australia’s Digital Health Sector Special Report 2021-22 [11] and informed the 2024 report [12]. The Census has ethics approval from the University of Tasmania and RMIT University Human Research Ethics Committee (project number 26607). It captures informed consent at the start of the census, including consent for the data to be available as per the Data Management and Access Policy [13].

The 2023 Census [14] was the third time the census had been held in Australia. It was held online from 1 July to 13 August 2023. This analysis examines the 2023 data as this was the first time explicit questions pertaining to gender were included. Due to the small number of respondents (*n* = 2) that identified as “Non-binary, gender-fluid, agender”, they have been excluded from the analysis to maintain their confidentiality. Any respondent who indicated they did not want to share how they identify their gender (*n* = 10) was also excluded from analysis. The analysis examined if there was a difference between women and men in the following items: (1). Education, (2) Professional development, (3). Occupation, (4). Workforce intention. A Pearson’s Chi-Square test of independence was conducted to examine the associations between gender and formal education, occupation, and the future workforce intentions of individuals within the Australian specialist digital health workforce. It was hypothesized that there would be significant associations between gender and the educational and occupational profile of people who self-identify as specialists in the Australian digital health workforce. Descriptive and inferential data analysis was undertaken using IBM SPSS Statistics (version 29.0.2.0) [15]. The significance level was set at *p* < 0.05.

## Results

### Demographics overview

The Census collected 857 valid responses, of which 595 (69.43%) respondents identified as woman, and 262 (30.57%) were men. At the time of taking the Census, all respondents were employed within Australia. 3.38% (29/857) of respondents identified as a First Nations person.

### Education and gender

**Fig. 1:**
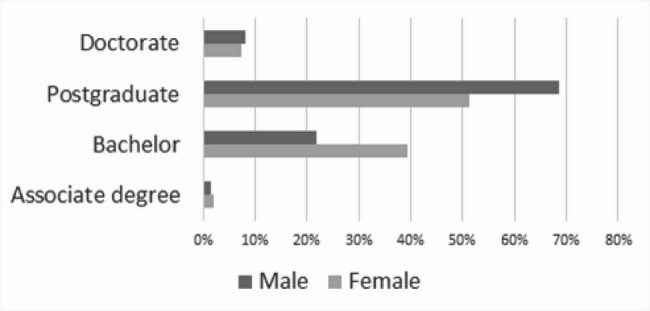
Type of formal digital health qualification and gender (*n* = 315; *p* < 0.001)

**Fig. 2:**
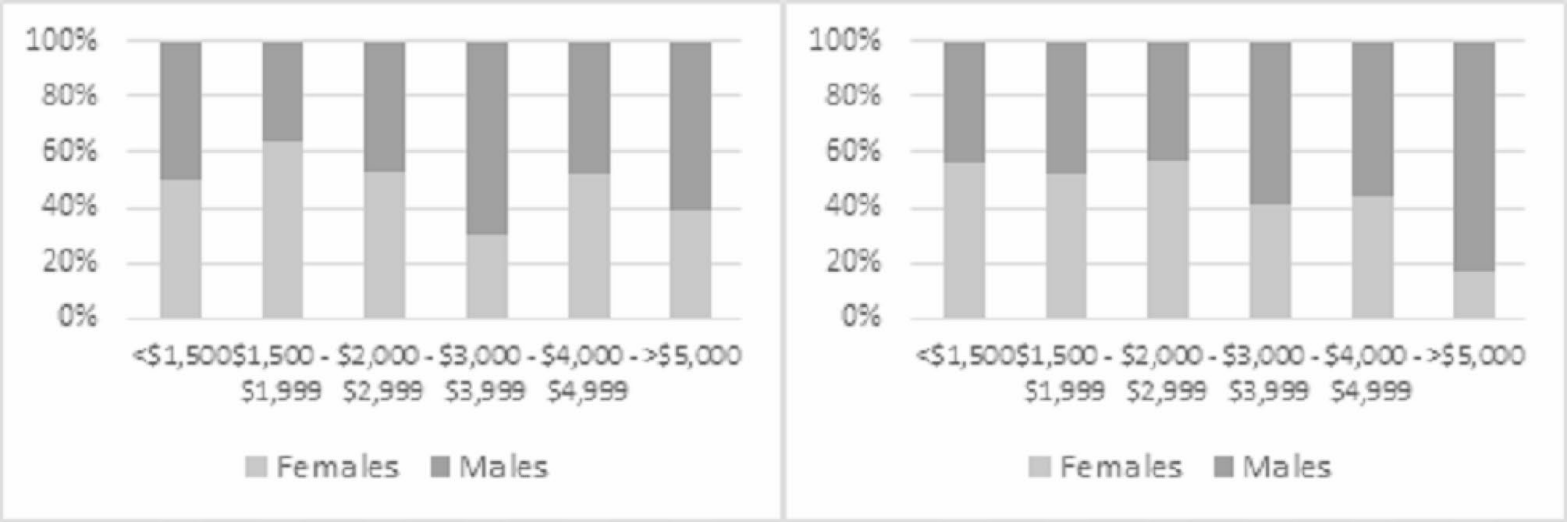
Remuneration and gender (left) (*n* = 272; *p* = 0.061) versus Remuneration and gender with a formal digital health qualification (right) (*n* = 272; *p* < 0.001)

The analysis identified there was a statistically significant association (*p* < 0.001) between gender and education (Table 1; Fig. 1). In women, there was a more equal distribution of those with and those without a formal digital health qualification in women than in men (*p* < 0.001). However, where the respondent holds a formal digital health qualification, men were more likely (*p* < 0.001) to have a postgraduate qualification than women, who had a higher proportion of bachelor level qualifications (Fig. 2).

There was an insufficient number of responses to be able to examine the correlation between gender and Indigenous status. A statistically significant association was identified between country of birth and gender where respondents reported a formal digital health qualification, whereby the majority of women with a formal qualification reported they were born in Australia (80.4%:19.6%) compared to men (54.5%:45.5%), indicating women with a formal qualification born overseas were less likely to be employed in a specialist digital health role compared to their male counterparts. No statistically significant difference was identified between those who identified as a First Nations person and gender, and separately people living with a disability and gender, in those who reported a formal digital health qualification, however this may be due to the small sample size in both diverse groups (< 10 respondents in each group). There was a statistically significant difference (*p* < 0.001) in remuneration and gender in those with a formal qualification, indicating that men with a formal qualification earnt more than women with a formal qualification in digital health. There was no difference when controlling for the type of qualification.

**Table 1:** Formal digital health (DH) qualification and gender

Topic	Categories	Women	Men	Significance
**Formal DH qualification** **(*n*** **= 790)**	Yes	250 (45.4%)	77 (32.2%)	*p* < 0.001
	No	301 (54.6%)	162 (67.8%)	
	Total	551	239	
**Type of DH qualification (*n*** **= 315)**	Associate degree	5 (2.1%)	1 (1.4%)	*p* < 0.001
	Bachelor (including honours)	95 (39.3%)	16 (21.9%)	
	Postgraduate	124 (51.2%)	50 (68.5%)	
	Doctorate	18 (7.4%)	6 (8.2%)	
	Total	242	73	
**Australian versus other country of birth & formal DH qualification (*n*** **= 329)**	Australian birth, formal DH qualification	201 (80.4%)	42 (54.5%)	*p* < 0.001
	Other country of birth, formal DH qualification	49 (19.6%)	35 (45.5%)	
	Total	250	77	
**Remuneration (*n*** **= 272)**	<$1,500	231 (41.8%)	102 (41.3%)	*p* = 0.061
	$1,500 - $1,999	83 (15.0%)	21 (8.5%)	
	$2,000 - $2,999	162 (29.3%)	63 (25.5%)	
	$3,000 - $3,999	38 (6.9%)	40 (16.2%)	
	$4,000 - $4,999	19 (3.4%)	8 (3.2%)	
	*≥*$5,000	19 (3.4%)	13 (5.3%)	
	Total	552	247	
**Remuneration with a formal qualification (*n*** **= 272)**	<$1,500	49 (23.6%)	12 (18.8%)	*p* < 0.001
	$1,500 - $1,999	35 (16.8%)	10 (15.6%)	
	$2,000 - $2,999	81 (38.9%)	19 (29.7%)	
	$3,000 - $3,999	29 (13.9%)	13 (20.3%)	
	$4,000 - $4,999	10 (4.8%)	4 (6.2%)	
	*≥*$5,000	4 (1.9%)	6 (9.4%)	
	Total	208	64	

### Professional development

Respondents could identify different types of professional development they undertook. An analysis of professional development type and gender identified there was a statistically significant difference in gender (*p* < 0.05), with women more likely to have attended a seminar, read a journal or news article related to digital health, or listened to digital health podcast, with nearing significance (*p* = 0.058) for conference attendance (Table 2). There was no statistical correlation (*p* = 0.237) between gender and undertaking digital health training that was not a formal qualification.

**Table 2:** Professional development activities already undertaken and gender (*n* = 857)

Categories (*n* = 857)	Response	Women (*n* = 595)	Men (*n* = 262)	Significance
Conference attendance	No	334 (39.0%)	138 (16.1%)	*p* = 0.058^
	Yes	261 (30.5%)	124 (14.5%)	
Seminar attendance	No	292 (34.1%)	149 (17.4%)	*p* = 0.048*
	Yes	303 (35.4%)	113 (13.2%)	
Training	No	443 (51.7%)	193 (22.5%)	*p* = 0.237
	Yes	152 (17.7%)	69 (8.1%)	
Read a journal	No	315 (36.8%)	136 (15.9%)	*p* = 0.049*
	Yes	280 (32.7%)	126 (14.7%)	
Read news related to digital health	No	282 (32.9%)	131 (15.3%)	*p* = 0.048*
	Yes	313 (36.5%)	131 (15.3%)	
Listened to digital health podcast	No	305 (35.6%)	127 (14.8%)	*p* = 0.047*
	Yes	290 (33.8%)	135 (15.8%)	

*Statistically significant, ^nearing significance

### Occupation

There was a statistically significant distribution of organisation type and gender (*p* < 0.001) (Table 3). Hospitals employed the largest percentage of both women and men. More men were employed at health technology organisations and state health departments compared to women. Women were more likely to be employed by larger organisations (over 1000 employees) compared to men. Men were more likely to work in organisations with less than 10 employees (16/180; 8.89%), 10–99 employees (24/180; 13.33%), or 100–499 employees (26/180; 14.44%) compared to women (*p* = 0.004) (Table 4).

**Table 3:** Organizational type where respondents are employed and gender (*n* = 618)

Categories (*n* = 618)	Women (*n* = 438)	Men (*n* = 180)	Significance
Community healthcare service	10 (2.28%)	2 (1.11%)	*P* < 0.001
Defence force/military	1 (0.23%)	1 (0.56%)	
Educational facility	31 (7.08%)	11 (6.11%)	
Federal health organisation	12 (2.74%)	12 (6.67%)	
Health technology organisation	44 (10.05%)	35 (19.44%)	
Hospital	199 (45.43%)	49 (27.22%)	
Indigenous health service	3 (0.68%)	2 (1.11%)	
Local health service/district/network	38 (8.68%)	14 (7.78%)	
Other not-for-profit organisation	17 (3.88%)	8 (4.44%)	
Other private organisation	14 (3.20%)	10 (5.56%)	
Other public/government organisation	11 (2.51%)	3 (1.67%)	
Primary care or primary health network	7 (1.60%)	4 (2.22%)	
Private practice	0 (0.00%)	3 (1.67%)	
State health department	51 (11.64%)	26 (14.44%)	

**Table 4:** Size of organizations respondents are employed by and gender (*n* = 617)

Categories (*n* = 617)	Women (*n* = 437)	Men (*n* = 180)	Significance
100–499	48 (10.98%)	26 (14.44%)	*P* = 0.004
10–99	42 (9.61%)	24 (13.33%)	
500–1000	41 (9.38%)	14 (7.78%)	
Less than 10	13 (2.97%)	16 (8.89%)	
More than 1000	293 (67.05%)	100 (55.56%)	

Majority of both women (341/438; 77.85%) and men (128/180; 71.11%) were employed in permanent positions. A higher percentage of men (10/180; 5.56%) were in casual positions compared to women (10/438; 2.28%), and self-employment was more common among men (9/180; 5.00%) than women (6/438; 1.37%) (*p* = 0.008). Women were more likely to report to other women (213/322; 66.15%) compared to men (53/110; 48.18%), and men were more likely to report to men (53/110; 48.18%) compared to women (105/322; 32.61%) (*p* = 0.006).

**Fig. 3:**
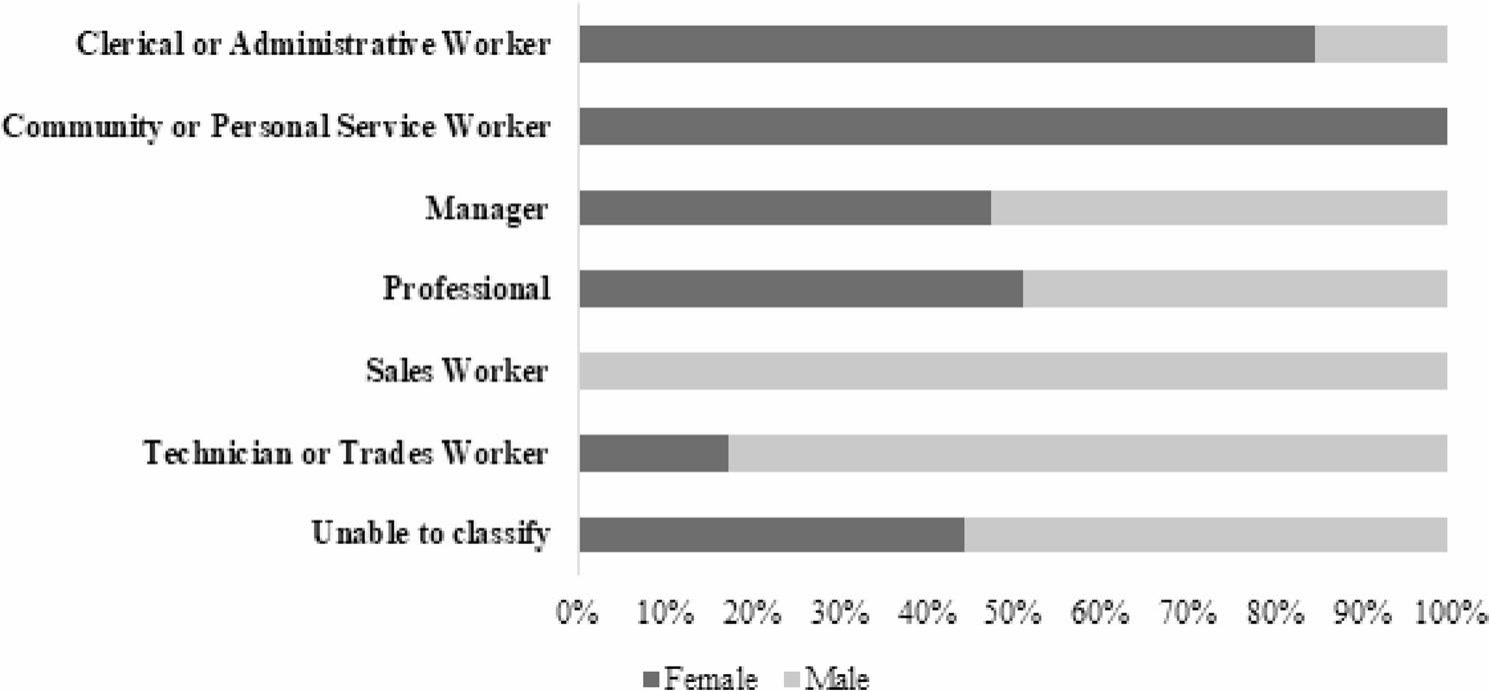
Respondent’s major group association and gender (*n* = 689; *p* = 0.02)

A significant association was found between gender and the major group a respondent was employed by (*p* = 0.020). Over half of both men and women identified as ‘professionals’. More men identified as ‘managers’ compared to women. No men categorised themselves as ‘community or personal service worker’, whilst no women categorised themselves as ‘sales workers’ (Fig. 3). There was no significant association observed between gender and how long an individual had been in their role (p = 0.781) or the specialist digital health workforce (p = 0.093).

Gender was significantly associated with an individual’s occupational specialty (*p* < 0.001) (Table 5). Women identified health information management, health librarianship, and health informatics as their top three occupational specialities. Men identified health informatics, health information technology, and health information management as their top three occupational specialities. Furthermore, more women occupied health librarianship (92/485, 18,97%) and clinical coding (29/485, 5.98%) roles compared to men (8/3.92% and 1.47% respectively). More men (79/240; 32.92%) were registered health practitioners compared to women (137/559; 24.51%), which was a statistically significant association (*p* = 0.014).

**Table 5:** Occupational specialty and gender (*n* = 689)

Categories (*n* = 689)	Women (*n* = 485)	Men (*n* = 204)	Significance
Biomedical engineering	1 (0.21%)	2 (0.98%)	*p* < 0.001
Clinical coding	29 (5.98%)	3 (1.47%)	
Clinical documentation improvement	16 (3.30%)	8 (3.92%)	
Epidemiology	3 (0.62%)	0 (0.00%)	
Health artificial intelligence	3 (0.62%)	4 (1.96%)	
Health cyber security	2 (0.41%)	2 (0.98%)	
Health data science/analytics	26 (5.36%)	14 (6.86%)	
Health informatics	79 (16.29%)	54 (26.47%)	
Health information management	109 (22.47%)	24 (11.76%)	
Health information technology	47 (9.69%)	44 (21.57%)	
Health innovation	32 (6.60%)	10 (4.90%)	
Health interoperability	13 (2.68%)	10 (4.90%)	
Health librarianship	92 (18.97%)	8 (3.92%)	
Health simulation	1 (0.21%)	1 (0.49%)	
Health technology assessment	3 (0.62%)	2 (0.98%)	
Translational bioinformatics	0 (0.00%)	2 (0.98%)	
Unable to classify	29 (5.98%)	16 (7.84%)	

**Fig. 4:**
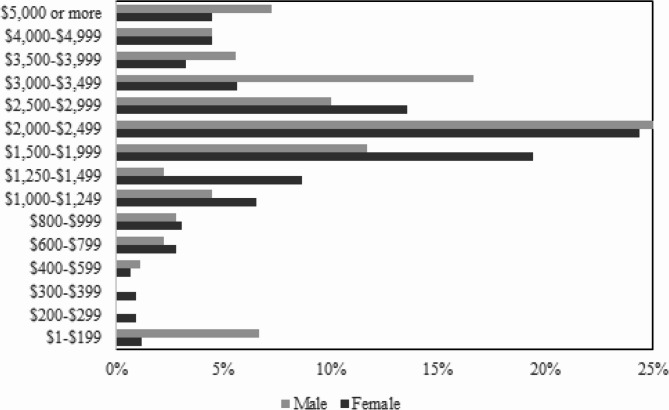
Remuneration and gender (*n* = 607; *p* < 0.001)

Gender was significantly associated with hours worked per week (*p* = 0.008), and there was a nearing significant association between gender and how many hours of work an individual was paid for per week (*p* = 0.087). There was also a significant association between gender and remuneration (*p* < 0.001) (Fig. 4). The remuneration range with the highest number of both men (45/180; 25.00%) and women (104/427; 24.36%) was $2,000-$2,499 per week. Women had a higher representation in the $1,500-$1,999 (83/427; 19.44%) range compared to men (21/180; 11.67%). Men had a higher representation in the $3,000-$3,499 range (30/180; 16.67%) compared to women (24/427; 5.62%). In the highest remuneration range ($5,000 or more), men (13/180; 7.22%) had a higher percentage compared to women (19/427; 4.45%).

Respondents were asked eight questions about their perceptions of their remuneration (Table 6). There was a significant association between gender and whether an individual perceived they received financial recognition based on their level of performance. A higher percentage of women disagreed (153/427; 35.83%) with the statement compared to men (37/180; 20.56%). Similarly, there was a significant association between gender and whether an individual believed that women and men are paid the same rates for performing similar work within their organisation. A higher percentage of women disagreed with the statement (110/427; 25.76%) compared to men (8/180; 4.44%).

**Table 6:** Gender-based analysis of employee perceptions on compensation and pay equity (*n* = 607)

Census section	Topic	*p* value
Section 5: Occupation and paid employment	Financial recognition based on performance	0.001*
	Fair compensation compared to similar work in my organization	0.093^
	Fair compensation compared to similar work in other organizations	0.960
	Salary reflects experience and skills	0.144
	Discussed pay with supervisor past 12 months	0.070^
	Understand pay and bonus decisions and processes	0.137
	Women and men paid same for similar work in my organization	< 0.001*
	Initial pay or remuneration offers are fair	0.282

*Statistically significant, ^nearing significance

### Future workforce intentions

Nearly half (83/176; 47.16%) of men expressed a desire to reach senior leadership positions, whereas less than a quarter (102/423; 24.11%) of women shared that ambition (*p* < 0.001). There was a significant association between gender and the factors that respondents considered important when considering a new job or career advancement (*p* < 0.001). Men placed slightly more importance on compensation (22/204; 10.78%) compared to women (43/483; 8.90%). Both women (119/483; 24.64%) and men (50/204; 24.51%) equally prioritised work/life balance, while women valued flexible work conditions (59/483; 12.22%) more than men (14/204; 6.86%).

**Fig. 5:**
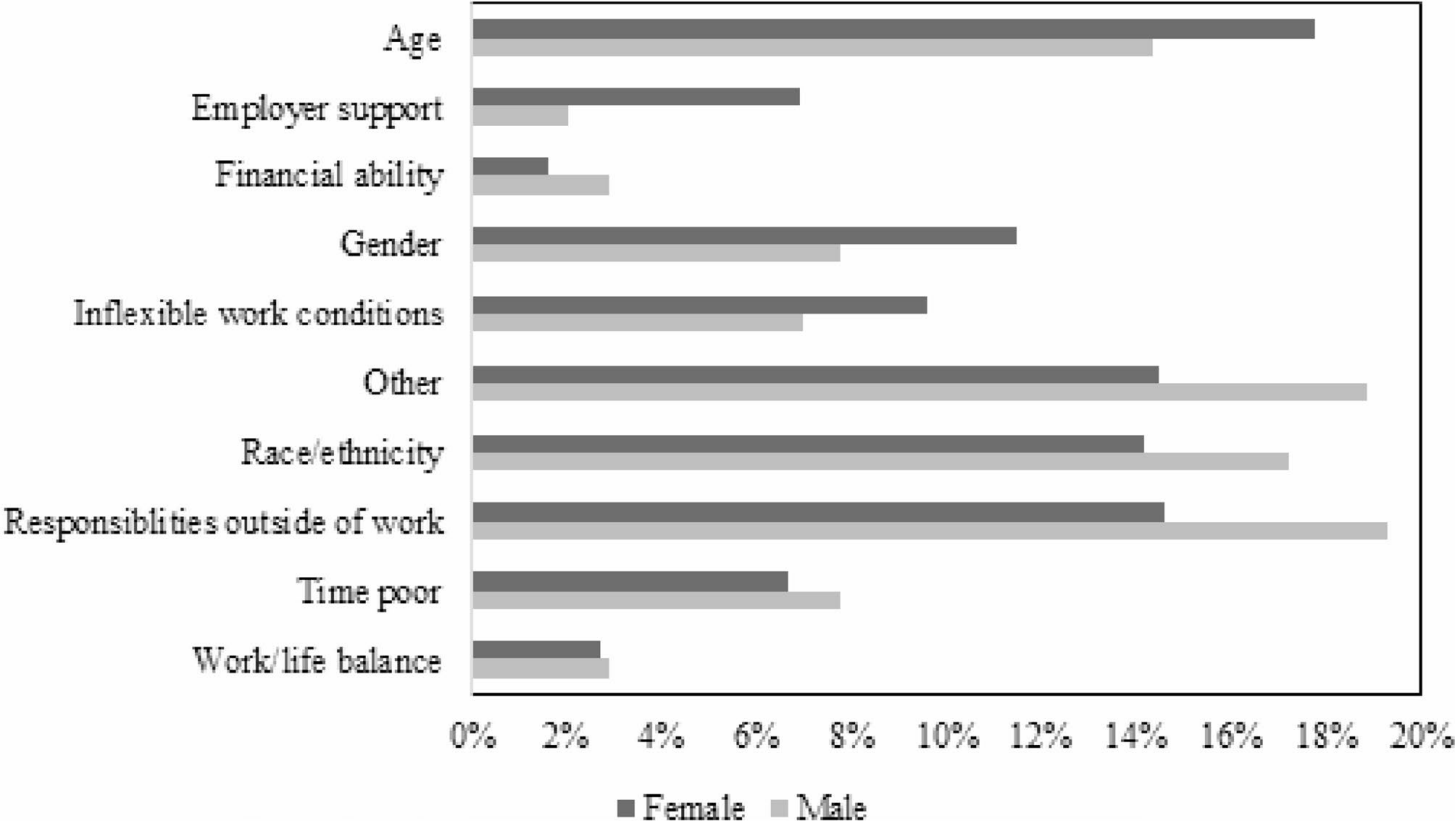
Perceived barriers to progression and gender (*n* = 363; *p* = 0.010)

There was a significant association between gender and what an individual perceived as a barrier to their career progression (*p* = 0.010) (Fig. 5). Age was perceived as a barrier by slightly more women than men. Gender was considered a barrier for more women compared to men. Financial constraints were considered a barrier for more women than men, while being time poor was perceived as a stronger barrier by men compared to women. Responsibilities outside of work were a barrier for more men, which was higher than women who felt the same. Men were more optimistic about their career growth prospects compared to women (*p* < 0.05). Similar proportions of men and women felt they needed to prove themselves within their workplaces, whilst a slightly higher percentage of men (30/107; 28.04%) do not feel that they need to prove themselves compared to women (58/323; 17.96%) (*p* = 0.031).

## Discussion

This study highlights that persistent gender disparities in the health workforce are translating into the specialist digital health workforce, revealing how structural, cultural, and organisational factors shape career trajectories and workforce intentions. By correlating gender data with educational attainment, professional development, job roles, and workforce intentions, the findings highlight gaps that demand attention and provide valuable insights for fostering gender equity within the sector.

The observed gender disparities, particularly in leadership, advanced qualifications, and remuneration, reflect entrenched patterns documented across the broader health and science, technology, engineering, and mathematics (STEM) sectors in Australia [16]. These disparities are not only a matter of representation but also signal deeper issues of access to opportunity, recognition, and organisational support [17]. For example, only 32.2% of women hold formal digital health qualifications compared to 45.4% of men (*p* < 0.001). Women also reported fewer chances for leadership training and advanced certification then their male counterparts, pointing to structural barriers in workplace learning pathways. The underrepresentation of women in technology-intensive and senior roles, despite their substantial presence in the overall health workforce, aligns with well-established evidence of vertical and horizontal gender segregation [16]. Specifically, women were more likely to occupy administrative or support role, while men predominated in leadership and technical domains such as health informatics and data analytics. This segregation is perpetuated by restrictive gender norms, limited access to high-visibility professional development, and persistent pay inequity, all of which are reinforced by organisational cultures and broader societal expectations [18]. Notably, only 25% of women surveyed aspired to senior leadership positions compared to 50% of men (*p* < 0.001), with many citing workplace discrimination and poor work-life balance as significant barriers to retention and progression. Importantly, the findings suggest that even when women attained formal digital health qualifications, they do not experience equitable career advancement or remuneration. This points to the inadequacy of focusing solely on educational attainment as a solution and highlights the need for systemic change in workplace practices, leadership pathways, and recognition systems [19].

These gaps have practical implications for workforce sustainability, innovation and health system effectiveness. Addressing gender inequity is not only a matter of social justice but also essential for harnessing the full potential of the digital health workforce as Australia responds to ongoing health system challenges [19]. Strategies should include mandating pay transparency to ensure equal pay for equal work, setting gender quotas for leadership and technical roles, expanding access to leadership development, mentorship, and sponsorship programs for women and other underrepresented groups, and embedding gender impact analysis in policy and program design.

Census findings support these recommendations, highlighting the importance of structured mentorship and inclusive professional networks in enabling women to navigate career progression more effectively. Fostering inclusive organisational cultures and promoting digital health career pathways to girls and women from an early age will be critical for improving representation and retention. These recommendations are consistent with national and international best practice and their implementation is supported by evidence that diverse and inclusive workforces lead to improved organisational performance and innovation [20, 21].

A strength of this study is its comprehensive, national scope and the use of a validated census tool, enabling robust benchmarking and the identification of actionable gaps. The large sample size enhances generalisability and provides a strong foundation for sector-wide reform. However, several limitations must be acknowledged. The cross-sectional design restricts causal inference, and the reliance on self-reported data introduces potential bias [22]. Due to confidentiality concerns associated with small sample sizes, the experiences of non-binary individuals, First Nations respondents, and respondents living with a disability could not be reported, limiting the study’s ability to fully address intersectional inequities. Future research should employ longitudinal and mixed method approaches to evaluate the impact of implemented strategies and capture the experiences of all gender identities, including those currently underrepresented in specialist digital health workforce data. Enhanced data collection on intersectionality will be essential for developing truly inclusive workforce policies.

## Data Availability

The datasets used and/or analysed during the current study are available from the corresponding author on reasonable request.

## Abbreviations

DH: Digital health
STEM: Science, technology, engineering and mathematics
